# Hyperphosphatemia as a potential risk factor for arteriovenous fistula dysfunction: A retrospective study in hemodialysis patients

**DOI:** 10.1371/journal.pone.0335599

**Published:** 2025-10-30

**Authors:** Yujie Jiang, Chengji Cui, He Nan, Tianying Chang, Fan Li, Shoulin Zhang

**Affiliations:** 1 College of Traditional Chinese Medicine, Changchun University of Chinese Medicine, Changchun, Jilin, China; 2 Department of Nephrology, The Affiliated Hospital of Changchun University of Chinese Medicine, Changchun, Jilin, China; Royal Holloway University of London, UNITED KINGDOM OF GREAT BRITAIN AND NORTHERN IRELAND

## Abstract

**Background:**

The study investigates the relationship between Hyperphosphatemia and arteriovenous fistula dysfunction in patients undergoing maintenance hemodialysis.

**Methods:**

Data were collected from patients who had their first arteriovenous fistula creation and regular maintenance hemodialysis between 2019 and 2023. Patients were divided into four groups based on serum phosphorus levels (<1.33 mmol/L, 1.33–1.61 mmol/L, 1.61–1.965 mmol/L, > 1.965 mmol/L). Statistical methods included Kaplan-Meier survival curves, Cox proportional hazards regression models, and Restricted Cubic Spline.

**Results:**

The study included 239 patients. Kaplan-Meier survival curves showed that Hyperphosphatemia significantly correlated with arteriovenous fistula dysfunction (P = 0.0052). Cox univariate analysis showed phosphorus (HR = 3.16, P < 0.001) is risk factor of arteriovenous fistula dysfuntion. Multivariate Cox regression analysis further confirmed high phosphorus levels were an independent risk factor of AVF dysfuntion (P for trend<0.001). Restricted Cubic Spline showed a nonlinear relationship between phosphorus levels and arteriovenous fistula dysfunction (P for non-linearity = 0.024), with a cutoff point at 1.544 mmol/L (LR = 0.035).

**Conclusion:**

Hyperphosphatemia is an independent risk factor for arteriovenous fistula dysfunction, emphasizing the need for clinical practice to pay attention to the impact of mineral metabolism disorders on the integrity of arteriovenous fistula and to strengthen phosphate management.

## Introduction

The prevalence of chronic kidney diseases has been increasing due to changes in people’s living environments and the development of population aging, becoming a major public health issue. When the disease reaches its terminal stage, most patients will rely on renal replacement therapies (hemodialysis, peritoneal dialysis, or kidney transplantation) to maintain their health. China currently has approximately 916,000 hemodialysis patients. However, Arteriovenous fistula (AVF), the recommended vascular access of hemodialysis [1], exhibits a 1-year patency rate of 62%−68% and a 2-year patency rate of 38%−56% [2],resulting from multiple factors including surgical trauma, repeated cannulation, suboptimal vascular conditions, oxidative stress, and infection. AVF dysfunction compromises adequate renal replacement therapy and severely impacts clinical outcomes. Repeated surgical interventions for AVF dysfunction may also lead to progressive depletion of vascular access resources.Consequently, identifying risk factors for AVF dysfunction and developing accessible biomarkers for risk prediction are critical to preserving vascular access integrity.

Elevated phosphorus levels induce osteogenic phenotypic transformation of vascular smooth muscle cells (VSMCs), exacerbate endothelial dysfunction, and promote oxidative stress-mediated vascular injury. In chronic kidney disease (CKD) patients, recent studies have established that phosphorus dysregulation is a pivotal driver of vascular calcification [3]. However, the threshold of serum phosphorus and its dose-response relationship with AVF dysfunction remain undefined.This retrospective cohort study systematically investigates the dose-response relationship between serum phosphorus levels and AVF dysfunction. Our findings suggest that conventional thresholds for phosphorus management may not represent safe thresholds, thereby providing novel insights into vascular access management and advocating for stricter serum phosphorus targets to mitigate clinical risks.

## Materials and methods

### Patients and data extraction

**Fig 1:**
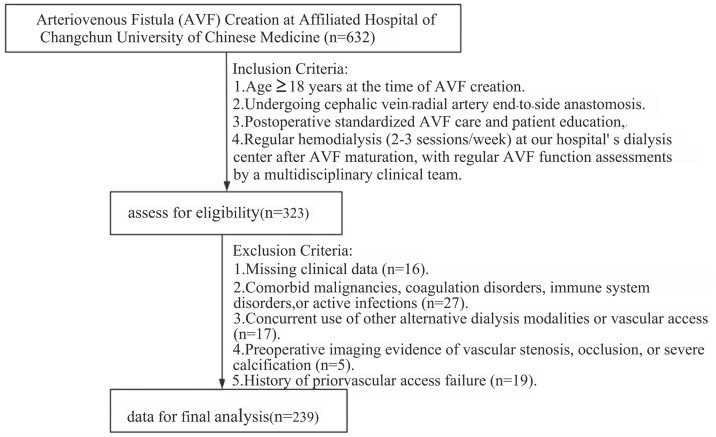
Recruitment and screening flowchart.

This retrospective cohort study enrolled 239 MHD patients who had AVF creation at Affiliated Hospital of Changchun University of Chinese Medicine from June 2019 to June 2023 (Fig 1). All participants provided written informed consent. All patient information is anonymized.

Baseline demographic characteristics and laboratory parameters were extracted from the hospital information system (HIS) and hemodialysis data platform. The demographic, clinical, and laboratory characteristics of the patients were collected at the time of AVF creation.Collected variables included demographics (sex, age, AVF creation date), past history (diabetes, hypertension, cardiovascular disease), laboratory parameters (complete blood count, serum calcium, parathyroid hormone (PTH), potassium, phosphorus, uric acid) and AVF patency.

Retrospective follow-up was conducted via electronic medical records and dialysis logs to document endpoint events, including AVF dysfunction, dialysis withdrawal, and death. Follow-up duration was recorded in months, with the study period concluding in December 2023.

Diagnosis can be made when meeting the following conditions [4,5]: (I) luminal stenosis >50% compared to adjacent normal vessels; (II) disappearance offistula murmur on clinical auscultation; (III) weakening or disappearance of pulse at the fistula; (IV) diagnosis of fistula thrombosis by ultrasound; and (V) blood flow <150 mL/min during dialysis.

### Statistical analysis

Data were analyzed using SPSS 23.0 and R 4.3.1. Continuous variables were assessed for normality via the Kolmogorov-Smirnov test. Normally distributed data are presented as mean ± standard deviation (SD), with group comparisons performed using ANOVA; non-normally distributed data are expressed as median (interquartile range, IQR) and analyzed via the Kruskal-Wallis H test. Categorical variables are reported as frequencies (percentages), with differences evaluated by χ² tests.

Kaplan-Meier curves were generated to compare AVF patency rates across groups, with log-rank tests for intergroup differences. Univariate Cox regression identified potential predictors of AVF dysfunction (P < 0.05 or clinically relevant variables), followed by multivariate Cox regression to adjust for confounders. E-values were calculated to quantify the strength of unmeasured confounding required to explain away the observed hazard ratio (HR). Restricted cubic splines (RCS) were used to model the nonlinear association between serum phosphorus and AVF dysfunction, identifying inflection points via LR (Likehood ratio) tests. Subgroup analysis were performed by sex, age (≥60 vs. < 60 years), diabetes, hypertension, and AVF vintage (≤9.5/9 ~ 17/17 ~ 28/ ≥ 28 months) to assess interaction effects.The proportional hazards assumption was checked using the Schoenfeld residuals.To address competing risks from events such as death, sensitivity analyses were performed using the Fine-Gray competing risk model. P value <0.05 was considered statistically significant.

As a retrospective observational study utilizing clinical data from the HIS and hemodialysis data platform, this research did not involve additional patient interventions or privacy breaches. In accordance with the Ethical Management Guidelines for Scientific Research at the Affiliated Hospital of Changchun University of Chinese Medicine and China’s Ethical Review Measures for Biomedical Research Involving Human Subjects, this study was exempt from institutional ethics review. All procedures adhered to the principles of the Declaration of Helsinki, with data fully de-identified to ensure patient confidentiality.

The datasets supporting this study are publicly accessible and have been archived in the Mendeley Data, available at: https://doi.org/10.17632/2m6w2dzn6w.1.

## Results

This study included 239 patients (Table 1).A mean age of patients was 58.5 ± 14.4 years. 152 (63.6%) patients were male. 87 (36.4%) patients were female.Past history was distributed as follows: diabetes (51.0%), hypertension (88.7%), and cardiovascular disease (CVD, 61.9%). Patients were divided into four groups based on serum phosphorus levels: T_1_ (<1.33 mmol/L, n = 57), T_2_ (1.33 ~ 1.61 mmol/L, n = 69), T_3_ (1.61 ~ 1.965 mmol/L, n = 53), and T_4_ (>1.965 mmol/L, n = 60). Intergroup analysis revealed significant differences in age, AVF vintage, PTH, phosphorus, uric acid levels and history of vitamin D analogs, CVD (all P < 0.05, Table 1). Additionally, T_4_ exhibited significantly shorter time of AVF dysfunction (23.8 ± 13.2 vs. 19.2 ± 11.1 vs.18.8 ± 11.7 vs. 15.4 ± 10.9, P = 0.002), with progressively elevated PTH (204.0 pg/mL vs. 262.2 pg/mL vs. 251.5 pg/mL vs. 338.3 pg/mL, P < 0.001) and uric acid levels (353.7 ± 124.1 μmol/L vs. 417.2 ± 109.0 μmol/L vs.443.4 ± 121.7 μmol/L vs. 495.8 ± 128.0 μmol/L, P < 0.01).

**Table 1:** Basic clinical data and biochemical parameters of the study groups according to phosphorus baseline.

Variables	Total (n = 239)	1 (n = 57)	2 (n = 69)	3 (n = 53)	4 (n = 60)	p
sex, n (%)						0.06
1 (female)	87 (36.4)	25 (43.9)	30 (43.5)	18 (34.0)	14 (23.3)	
2 (male)	152 (63.6)	32 (56.1)	39 (56.5)	35 (66.0)	46 (76.7)	
age, Mean ± SD	58.5 ± 14.4	62.8 ± 13.8	58.3 ± 13.2	61.2 ± 14.9	52.5 ± 14.1	< 0.001
AVF dysfunction, n (%)						0.313
0	105 (43.9)	26 (45.6)	36 (52.2)	20 (37.7)	23 (38.3)	
1	134 (56.1)	31 (54.4)	33 (47.8)	33 (62.3)	37 (61.7)	
diabetes, n (%)						0.258
0	117 (49.0)	32 (56.1)	29 (42.0)	23 (43.4)	33 (55.0)	
1	122 (51.0)	25 (43.9)	40 (58.0)	30 (56.6)	27 (45.0)	
hypertension, n (%)						0.129
0	27 (11.3)	11 (19.3)	8 (11.6)	4 (7.5)	4 (6.7)	
1	212 (88.7)	46 (80.7)	61 (88.4)	49 (92.5)	56 (93.3)	
CVD, n (%)						0.017
0	91 (38.1)	31 (54.4)	23 (33.3)	14 (26.4)	23 (38.3)	
1	148 (61.9)	26 (45.6)	46 (66.7)	39 (73.6)	37 (61.7)	
AVF vintage, month,Mean ± SD	19.3 ± 12.0	23.8 ± 13.2	19.2 ± 11.1	18.8 ± 11.7	15.4 ± 10.9	0.002
smoke, n (%)						0.07
0	195 (81.6)	48 (84.2)	56 (81.2)	48 (90.6)	43 (71.7)	
1	44 (18.4)	9 (15.8)	13 (18.8)	5 (9.4)	17 (28.3)	
Statins, n (%)						0.382
0	182 (76.2)	46 (80.7)	48 (69.6)	43 (81.1)	45 (75.0)	
1	57 (23.8)	11 (19.3)	21 (30.4)	10 (18.9)	15 (25.0)	
phosphate binders, n (%)						0.312
0	119 (49.8)	32 (56.1)	36 (52.2)	27 (50.9)	24 (40)	
1 (Sevelamer carbonate)	117 (49.0)	25 (43.9)	31 (44.9)	25 (47.2)	36 (60)	
2 (Lanthanum carbonate)	3 (1.3)	0 (0)	2 (2.9)	1 (1.9)	0 (0)	
vitamin D analogs, n (%)						0.008
0	56 (23.4)	18 (31.6)	15 (21.7)	16 (30.2)	7 (11.7)	
1 (Calcitriol)	106 (44.4)	23 (40.4)	32 (46.4)	24 (45.3)	27 (45.0)	
2 (Alfacalcidol)	33 (13.8)	8 (14.0)	15 (21.7)	4 (7.5)	6 (10.0)	
3 (Paricalcitol)	44 (18.4)	8 (14.0)	7 (10.1)	9 (17.0)	20 (33.3)	
anticoagulants, n (%)						0.419
0	181 (75.7)	48 (84.2)	51 (73.9)	35 (66.0)	47 (78.3)	
1 (Rivaroxaban)	14 (5.9)	3 (5.3)	5 (7.2)	4 (7.5)	2 (3.3)	
2 (Aspirin)	15 (6.3)	1 (1.8)	6 (8.7)	3 (5.7)	5 (8.3)	
3 (Clopidogrel)	10 (4.2)	1 (1.8)	2 (2.9)	6 (11.3)	1 (1.7)	
4 (Ticagrelor)	19 (7.9)	4 (7)	5 (7.2)	5 (9.4)	5 (8.3)	
WBC, *10^9^/L,Mean ± SD	7.8 ± 3.6	7.7 ± 5.2	7.9 ± 3.0	7.9 ± 3.1	7.7 ± 2.8	0.98
monocyte count, *10^9^/L,Mean ± SD	0.5 ± 0.2	0.5 ± 0.3	0.5 ± 0.3	0.5 ± 0.2	0.5 ± 0.2	0.552
RBC, *10^12^/L,Mean ± SD	3.4 ± 0.7	3.3 ± 0.7	3.5 ± 0.6	3.4 ± 0.6	3.2 ± 0.8	0.149
PTH, pg/ml,Median (IQR)	256.7 (151.3, 416.0)	204.0 (106.0, 305.8)	262.2 (147.3, 395.5)	251.5 (177.5, 354.8)	338.3 (207.3, 547.4)	< 0.001
lymphocyte count, *10^9^/L,Mean ± SD	1.4 ± 0.6	1.3 ± 0.7	1.4 ± 0.5	1.3 ± 0.6	1.4 ± 0.5	0.836
phosphorus, mmol/L,Mean ± SD	1.7 ± 0.5	1.1 ± 0.2	1.5 ± 0.1	1.8 ± 0.1	2.4 ± 0.4	< 0.001
calcium, mmol/L,Mean ± SD	2.1 ± 0.2	2.0 ± 0.3	2.1 ± 0.2	2.1 ± 0.2	2.0 ± 0.3	0.592
uric acid, μmol/L,Mean ± SD	427.6 ± 129.9	353.7 ± 124.1	417.2 ± 109.0	443.4 ± 121.7	495.8 ± 128.0	< 0.001
haemoglobin, g/L,Mean ± SD	98.3 ± 19.2	97.0 ± 19.9	101.0 ± 17.5	100.2 ± 19.3	94.9 ± 20.2	0.263
platelet, *10^9^/L,Mean ± SD	207.5 ± 85.0	197.1 ± 81.4	223.4 ± 83.1	203.9 ± 71.4	202.1 ± 99.7	0.309
neutrophil count, *10^9^/L,Mean ± SD	5.9 ± 3.5	5.9 ± 5.1	5.9 ± 2.6	5.9 ± 3.2	5.7 ± 2.6	0.986

CVD, cardiovascular disease. WBC, white blood cell. RBC, red blood cell. PTH,parathyroid hormone. AVF, arteriovenous fistula.

**Fig 2:**
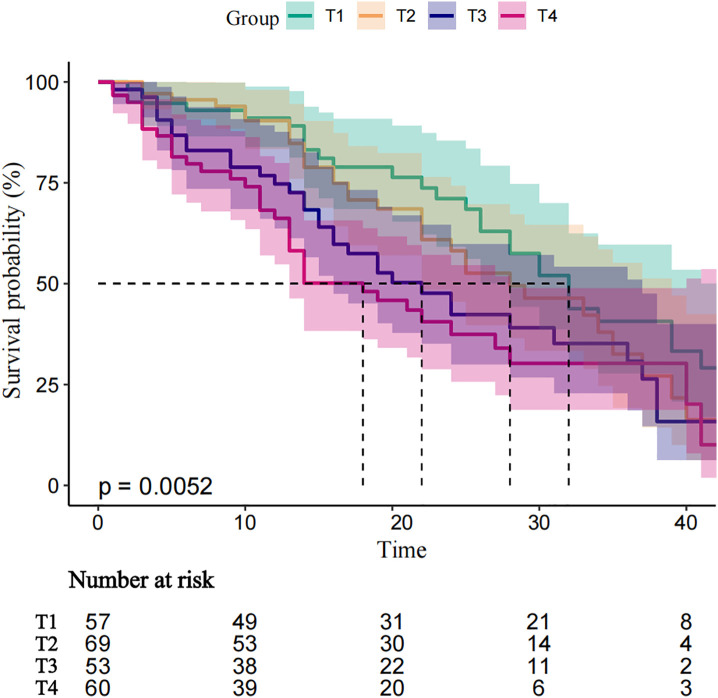
Kaplan-Meier analysis for AVF dysfunction events according to baseline of phosphorus.

During a median follow-up of 20 months, 134 patients (56.07%) experienced AVF dysfunction. Stenosis was the predominant subtype (n = 96, 40.17%), followed by thrombosis (n = 21, 8.79%) and low flow (n = 17, 7.11%). Cumulative patency rates at 6, 12, and 24 months were 88.28%, 82.01%, and 60.67%, respectively. Three deaths and four patients transferred to other hospitals for dialysis occurred during follow-up. Kaplan-Meier curves demonstrated a significant positive correlation between serum phosphorus levels and AVF dysfunction (log-rank P = 0.0052, Fig 2).

T_1_ (<1.33 mmol/L) 、T_2_ (1.33–1.61 mmol/L) , T_3_ (1.33–1.61 mmol/L) , T_4_ (>1.965 mmol/L) . AVF, arteriovenous fistula.

Univariate Cox regression identified serum calcium (HR = 0.26, 95% CI: 0.15 ~ 0.45; P < 0.001), phosphate (HR = 3.16, 95% CI: 2.63 ~ 3.80; P < 0.001), PTH (HR = 1.0007, 95% CI: 1.0003 ~ 1.0011; P < 0.01), and uric acid (HR = 1.0029, 95% CI: 1.0019 ~ 1.0039; P < 0.001) as significant predictors of AVF dysfunction. (Table 2). Each 1 mmol/L increment in phosphate was associated with a 3.16-fold increased risk of AVF dysfunction (HR = 3.16, 95% CI: 2.63 ~ 3.80; P < 0.001). Per 1 pg/mL increase in PTH conferred a 0.07% increased risk (HR = 1.0007, 95% CI: 1.0003 ~ 1.0011; P < 0.01), while per 1 μmol/L increase in uric acid elevated the risk by 0.29% (HR = 1.0029, 95% CI: 1.0019 ~ 1.0039; P < 0.001). The univariate Cox regression suggested calcium as a potential “protective” factor against AVF dysfunction. To more accurately assess the independent effect of calcium on AVF dysfunction, we performed a multivariate Cox regression adjusted for confounding factors. The results demonstrated that higher calcium (≥ 2.52 mmol/L) was not independently associated with AVF dysfunction (P > 0.05), indicating that the observed “protective” effect was primarily driven by confounding factors (Table 1S in S1 Table). RCS revealed a overall association between calcium and AVF dysfunction (P for overall<0.05). The test for non-linearity indicated a borderline significant association (P for non-linearity = 0.051, S1 Fig). To further explore the borderline significant association inflection in the RCS curve, we conducted a multivariate Cox regression using the data-driven cut-off of 2.09 mmol/L (Table 2S in S2 Table). The analysis indicated that patients with serum calcium levels ≥ 2.09 mmol/L had a higher risk of AVF dysfunction (HR = 0.39, 95% CI: 0.29 ~ 0.53, P < 0.001).

**Table 2:** Results of univariate Cox regression analysis of influencing factors of arteriovenous fistula dysfunction in patients.

Item	HR(95%CI)	P
Sex: man vs female	1.28 (0.96,1.72)	0.097
age	0.99 (0.98,1)	0.019
diabetes: 1 vs 0	0.89 (0.67,1.19)	0.43
hypertension: 1 vs 0	1.08 (0.67,1.76)	0.745
CVD: 1 vs 0	0.9 (0.68,1.2)	0.467
smoke: 1 vs 0	1.42 (0.96,2.1)	0.082
vitamin D analogs: ref. = 0		
1(Calcitriol)	0.93 (0.52,1.67)	0.82
2(Alfacalcidol)	0.66 (0.33,1.34)	0.252
3(Paricalcitol)	1.24 (0.71,2.18)	0.444
phosphate binders: ref. = 0		
1(Sevelamer carbonate)	1.2 (0.9,1.6)	0.224
2(Lanthanum carbonate)	1.64 (0.76,3.56)	0.208
Statins: 1 vs 0	1.11 (0.79,1.57)	0.532
anticoagulants: ref. = 0		
1(Rivaroxaban)	1.36 (0.69,2.66)	0.375
2(Aspirin)	0.8 (0.41,1.56)	0.505
3(Clopidogrel)	1.89 (0.83,4.27)	0.128
4(Ticagrelor)	1.33 (0.74,2.39)	0.346
WBC	1.02 (0.98,1.06)	0.404
monocyte count	0.76 (0.42,1.4)	0.379
calcium	0.26 (0.15,0.45)	< 0.001
RBC	1.12 (0.91,1.36)	0.281
PTH	1.0007 (1.0003,1.0011)	< 0.001
lymphocyte count	1.2 (0.95,1.51)	0.119
phosphorus	3.16 (2.63,3.8)	< 0.001
uric acid	1.0029 (1.0019,1.0039)	< 0.001
haemoglobin	0.9994 (0.9927,1.0062)	0.873
platelet	1.0009 (0.9992,1.0027)	0.301
neutrophil count	1.0085 (0.9684,1.0504)	0.681

CVD, cardiovascular disease. WBC, white blood cell. RBC, red blood cell. PTH,parathyroid hormone. AVF, arteriovenous fistula.

Multivariate Cox regression showed that T_4_ exhibited the highest risk (HR = 3.95, 95% CI: 2.42 ~ 6.47), significantly exceeding T_3_ (HR = 1.26, 95% CI: 0.79 ~ 2) and T_2_ (HR = 0.83, 95% CI: 0.49 ~ 1.39). Four adjusted models were constructed to evaluate the independent association between phosphorus levels and AVF dysfunction. After full adjustment (Model 4), the HRs (95% CI) from the lowest to highest phosphorus quartiles were 1.00 (reference), 0.83 (0.49 ~ 1.39), 1.26 (0.79 ~ 2), and 3.95 (2.42 ~ 6.47), confirming hyperphosphatemia as an independent risk factor (Table 3).

**Table 3:** Hazard ratio for arteriovenous fistula dysfunction events according to the baseline of phosphorus.

Models	T_1_	T_2_	T_3_	T_4_	P for trend
Unadjusted	reference	0.84 (0.51 ~ 1.37)	1.24 (0.81 ~ 1.9)	4.85 (3.23 ~ 7.29)	<0.001
Model 1	reference	0.83 (0.51 ~ 1.37)	1.2 (0.78 ~ 1.85)	4.67 (3.09 ~ 7.05)	<0.001
Model 2	reference	0.8 (0.48 ~ 1.32)	1.13 (0.72 ~ 1.77)	3.67 (2.31 ~ 5.86)	<0.001
Model 3	reference	0.79 (0.48 ~ 1.31)	1.13 (0.72 ~ 1.76)	3.6 (2.26 ~ 5.75)	<0.001
Model 4	reference	0.83 (0.49 ~ 1.39)	1.26 (0.79 ~ 2)	3.95 (2.42 ~ 6.47)	<0.001

Model 1: Adjusted for age and sex.; Model 2: Adjusted for variables in Model 1 plus uric acid, calcium, and parathyroid hormone.;Model 3: Adjusted for variables in Model 2 plus history of smoke. Model 4: Adjusted for variables in Model 3 plus history of hypertension, diabetes, white blood cell count, monocyte count, lymphocyte count, hemoglobin, and neutrophil count, use of Statins and anticoagulants.

**Fig 3:**
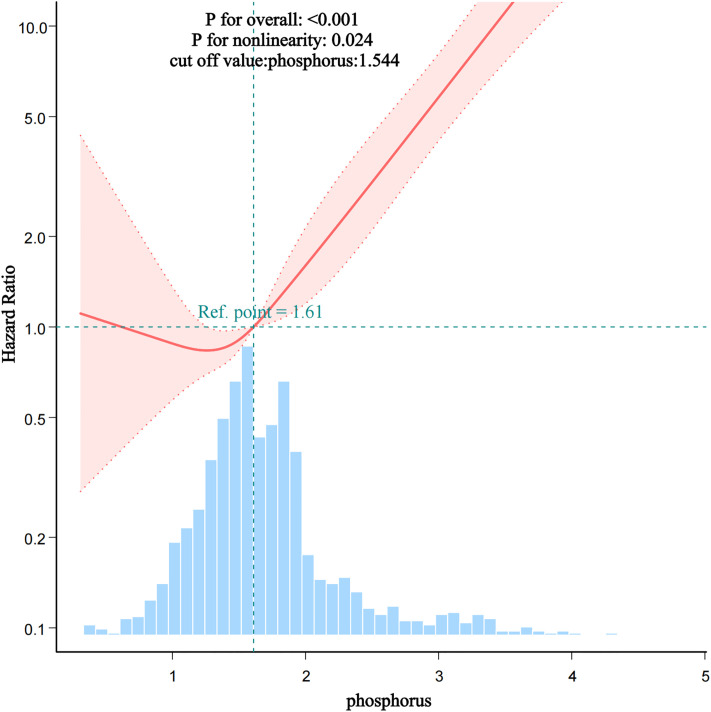
Restricted cubic spline (RCS) curve illustrating the relationship between serum phosphorus levels and AVF dysfunction.

Restricted cubic spline (RCS) analysis revealed a J-shaped nonlinear relationship between phosphorus level and AVF dysfunction, with a cutoff point at 1.544 mmol/L (LR = 0.035, Fig 3). Above this threshold, each 1 mmol/L increase in phosphorus elevated the risk of AVF dysfunction by 3.967 fold (HR = 3.967, 95% CI: 3.067 ~ 5.132, P < 0.001).

The solid red line represents the hazard ratio (HR), and the shaded area indicates the 95% confidence interval.

**Fig 4:**
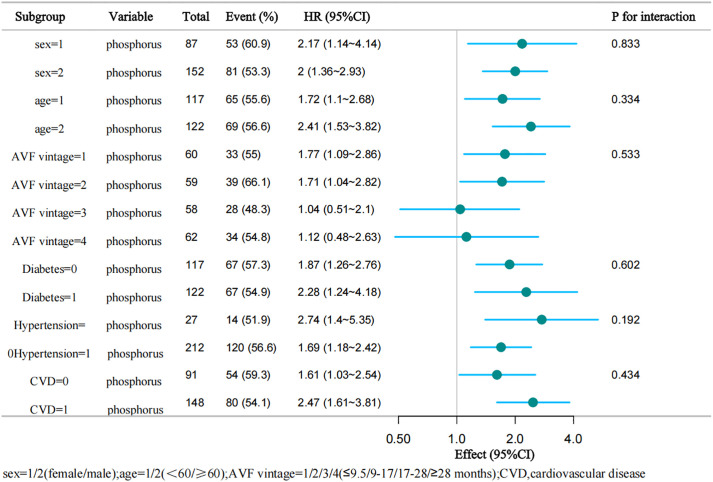
Subgroup analysis of the association between serum phosphorus levels and AVF dysfunction.

Subgroup analysis stratified by sex (female/male), age (<60 years/ ≥ 60), diabetes (yes/no), hypertension (yes/no),CVD (yes/no) and AVF vintage (≤9.5/9 ~ 17/17 ~ 28/ ≥ 28 months) demonstrated robust consistency across populations (P for interaction > 0.05 for all subgroup, Fig 4).

The calculated E-value of 6.47 supports the robustness of the study conclusions against potential unmeasured confounding. The Schoenfeld residual analysis revealed that all covariates exhibited P-values exceeding 0.05, indicating no statistically significant association between their residuals and time. Adequate adherence to the PH assumption across the entire model framework (global P = 0.366).The model indicated no significant competing risk effect (P = 0.215), with consistent directionality of the exposure-outcome association compared to the primary Cox analysis.

## Discussion

Kidney is one of the primary organs responsible for phosphorus clearance. In early-stage chronic kidney disease, compensatory increases in parathyroid hormone (PTH) and fibroblast growth factor-23 (FGF-23) levels maintain serum phosphate within normal ranges by enhancing renal excretion [6,7]. However, as renal function progressively declines, these compensatory mechanisms become inadequate, leading to hyperphosphatemia [8]. To manage concomitant chronic kidney disease-mineral and bone disorder (CKD-MBD), patients often receive long-term activated vitamin D therapy, which inadvertently promotes intestinal phosphate absorption and exacerbates hyperphosphatemia. Additional contributors include age, excessive dietary phosphate intake, suboptimal medication regimens, and dialysis-related factor [9,10]. To ensure optimal dialysis quality, we should monitor spKt/V, URR, dry weight, dialyzer phosphate clearance performance [11,12]. Notably, a nationwide Chinese study reported that only 26.7% of dialysis patients achieved target serum phosphate levels [13]. Critically, hyperphosphatemia is not merely an independent risk factor for cardiovascular mortality, it directly impairs vascular through induced vascular calcification and endothelial injury. This provides the mechanistic foundation for our investigation into the association between phosphorus and AVF dysfunction [14].

Vascular calcification in CKD patients has garnered significant clinical attention. A multicenter, prospective cohort study in China (n = 1,489) revealed that the prevalence of vascular calcification in dialysis patients reached 90.7% [15]. Currently recognized pathological mechanisms underlying AVF dysfunction include high-flow hemodynamics-induced vascular wall stress, surgical endothelial injury, localized inflammation from repeated cannulation, and CKD-associated chronic low-grade inflammation [16]. Therefore, identifying cost-effective biomarkers and optimizing modifiable risk factors, such as stringent phosphorus control and aggressive management of secondary hyperparathyroidism (SHPT), are critical strategies to preserve AVF patency.

Previous studies have established a robust association between hyperphosphatemia and AVF dysfunction [17,18]. This study is the first to demonstrate a J-shaped dose-response relationship between serum phosphorus and AVF dysfunction, with a cutoff point at 1.544 mmol/L. Above this threshold, each 1 mmol/L increase in phosphorus elevated the risk of AVF dysfunction by 3.967-fold (HR = 3.967, 95% CI: 3.067 ~ 5.132). Notably, even within the conventional “normal” phosphorus range, AVF dysfunction risk persists, underscoring the need to adopt stricter phosphorus targets rather than adhering to traditional thresholds. Furthermore, calcium dysregulation and SHPT were associated with AVF dysfunction, highlighting the imperative for comprehensive management of CKD-MBD.

In the multivariate Cox regression, we observed a noteworthy phenomenon: the association between calcium and AVF dysfunction was highly sensitive to the chosen cut-off value. No significant association was found when using the upper limit of the normal range (2.52 mmol/L). However, when a statistically derived cut-off (2.09 mmol/L) was applied, calcium levels above this value appeared to exhibit a “protective effect”.This apparent discrepancy is likely attributable to confounding. Serum calcium levels below 2.09 mmol/L are closely linked to pathological states such as hypoalbuminemia and disordered mineral metabolism due to poorly managed CKD-MBD, which contributes to vascular dysfunction. Therefore, patients with calcium levels above 2.09 mmol/L exhibit a significantly lower risk when compared to this high-risk, multimorbid “low-calcium” subgroup. This difference more likely reflects underlying disparities in nutritional status and CKD-MBD-related vascular health rather than a direct physiological effect of calcium ions. We caution that this likely reflects reference group distortion, as the comparator group is at high risk due to confounding comorbidities, rather than a true biological effect of calcium.

The pathogenesis of vascular calcification is closely linked to phosphorus metabolism dysregulation in CKD patients [19–22]. Hyperphosphatemia activates multiple signaling pathways, including NF-κB, Wnt/β-catenin, and BMP-2/Smad, inducing osteogenic phenotypic transformation of vascular smooth muscle cells (VSMCs) [23]. This process is accompanied by aberrant expression of osteogenic transcription factors, directly driving VSMC transition from a contractile to an osteoblast-like phenotype [24,25]. Elevated phosphate further exacerbates vascular calcification and endothelial dysfunction via the FGF23/Klotho axis and PTH-mediated pathways [26]. Beyond calcification, hyperphosphatemia contributes to endothelial dysfunction through oxidative stress and nitric oxide (NO) imbalance—key mechanisms of phosphorus-induced endothelial injury [27–29]. High phosphorus environments also accelerate endothelial damage by promoting apoptosis and autophagy. Notably, this study underscoring the need for stricter phosphorus targets (<1.544 mmol/L). Previous studies have identified significant correlations between normol serum phosphorus levels and endothelial dysfunction in hypertensive patients [30]. Furthermore, even within the conventional normal range, dietary phosphorus overload transiently elevates postprandial serum phosphorus, with acute exposure sufficient to impair endothelial-dependent vasodilation [28,31]. Remarkably, a single high-phosphorus meal can induce endothelial dysfunction, highlighting the clinical imperative to address occult phosphorus exposure and refine dietary management strategies. In this study, serum phosphorus levels were measured non-fasting immediately before dialysis sessions. Pre-dialysis phosphorus reflects cumulative phosphorus burden during the interdialytic period, which is mechanistically linked to long-term pathological processes such as vascular calcification and endothelial dysfunction. Importantly, pre-dialysis phosphorus serves as the primary clinical intervention target in routine mineral metabolism management, thereby enhancing its clinical relevance and academic significance for outcome interpretation.

Hyperphosphatemia has been implicated in the pathogenesis of thrombosis. Platelet-derived extracellular polyphosphate (PolyP), a pro-thrombotic linear chain of inorganic phosphate, is stored in platelet dense granuless [32]. Elevated extracellular inorganic phosphate levels have been shown to upregulate PolyP content within platelets, a process mediated by phosphate transporters, IP6K and V-type ATPases [33]. This suggests that hyperphosphatemia in patients may similarly enhance platelet PolyP levels, thereby predisposing to a pro-thrombotic state [34]. Hyperphosphatemia concurrently impairs vascular homeostasis by suppressing eNOS activity, thereby reducing bioavailability of nitric oxide. The deficiency of the vasodilator and platelet aggregation inhibitor promotes a pro-thrombotic, vasoconstrictive state [35]. Finally, as discussed earlier, hyperphosphatemia is a risk factor of vascular calcification. The calcified vascular wall not only loses compliance and disrupts hemodynamics but also provides a roughened, pro-adhesive surface that facilitates platelet attachment and thrombus formation [25].

SHPT is a critical driver of vascular calcification progression in CKD patients, and targeted interventions for SHPT may mitigate calcification and reduce vascular burden [36,37]. A series of in vivo and in vitro experiments have demonstrated that PTH contributes to vascular calcification through mechanisms independent of hyperphosphatemia [38,39]. PTH promotes osteogenic differentiation of endothelial cells via activation of extracellular signal-regulated kinase (ERK) and NF-κB signaling pathways [40]. In vitro studies using human aortic smooth muscle cells (HASMCs) exposed to varying PTH concentrations revealed that PTH downregulates histone deacetylase Sirtuin1 (SIRT1), induces apoptosis, and suppresses B-cell lymphoma-2 (Bcl-2) expression, thereby accelerating apoptosis [41]. Hypercalcemia exacerbates vascular calcification by stimulating vascular smooth muscle cells (VSMCs) to release matrix vesicles [22]. Under normophosphatemic conditions, elevated calcium concentrations significantly enhance VSMC calcification [42]. Urate crystal deposition directly injures vascular endothelium [43] and triggers inflammatory cascades, including release pro-inflammatory cytokines [44–46]. Furthermore, hyperuricemia suppresses vascular endothelial growth factor-A (VEGFA) by negatively regulating miR-92a, thereby increasing Kruppel-like factor 2 (KLF2) expression, which impairs endothelial cell proliferation and repair capacity [47]. Serum uric acid levels inversely correlate with nitric oxide [48]. As a pivotal vasodilator, NO deficiency compromises endothelial-dependent vasodilation and attenuates antiplatelet aggregation, ultimately predisposing to thrombus formation and luminal stenosis.

This single-center retrospective cohort study has several limitations. The modest sample size may introduce selection bias and limit statistical power. This study was conducted using electronic health records. Serum biomarkers including fibroblast growth factor-23 (FGF-23), Klotho, and interleukin-6 (IL-6) are not routinely measured in our clinical protocol. Consequently, systematic acquisition of these data was not feasible within the retrospective design framework.

This study establishes a clinically significant association between serum phosphorus and AVF dysfunction. However, as an observational study, it cannot definitively elucidate the underlying pathophysiology, such as the specific mechanisms by which hyperphosphatemia promotes thrombosis. Hyperphosphatemia is influenced by variables not fully captured here, including dietary phosphate intake and dialysis efficiency. Future research should therefore integrate extended follow-up with detailed biological markers (e.g., FGF-23, Klotho) and vascular imaging, while also meticulously tracking diet and dialysis parameters to adjust for these confounders.

## Conclusions

This retrospective cohort study is the first to demonstrate a J-shaped dose-response relationship between serum phosphorus levels and AVF dysfunction in MHD patients, identifying a critical phosphorus threshold of 1.544 mmol/L. When serum phosphorus exceeds this threshold, each 1 mmol/L increase elevates AVF dysfunction risk by 3.967-fold (HR = 3.967, 95% CI: 3.067 ~ 5.132). These findings challenge the conventional safety assumptions of phosphorus “normal ranges” and underscore the necessity for refined phosphorus management strategies, particularly in high-hemodynamic vascular access sites like AVF.

Clinically, serum phosphorus >1.544 mmol/L may serve as an early warning biomarker for AVF dysfunction. Integrating dynamic monitoring of PTH, uric acid, and vascular calcification scores could enhance risk stratification precision. Furthermore, calcium dysregulation, SHPT, and hyperuricemia were associated with AVF dysfunction, highlighting the systemic vascular injury effects of CKD-MBD. These results advocate for comprehensive management of CKD-MBD to mitigate multifactorial vascular compromise.

## Supporting information

S1 Fig Restricted cubic spline (RCS) curve illustrating the relationship between serum calcium levels and AVF dysfunction. The solid red line represents the hazard ratio (HR), and the shaded area indicates the 95% confidence interval.
(TIF)

S1 Table Hazard ratio for arteriovenous fistula dysfunction events according to the baseline of calcium. Model 1: Unadjusted.; Model 2: Adjusted for sex, age, history of hypertension, diabetes, cardiovascular disease, parathyroid hormone, phosphorus, use of phosphate binders, vitamin D analogs.;Model 3: Adjusted for variables in Model 2 plus uric acid, platelet. Model 4: Adjusted for variables in Model 3 plus white blood cell.
(DOCX)

S2 Table Hazard ratio for arteriovenous fistula dysfunction events according to the baseline of calcium(cut-off 2.09 mmol/L). Model 1: Unadjusted.; Model 2: Adjusted for sex, age, history of hypertension, diabetes, cardiovascular disease, parathyroid hormone, phosphorus, use of phosphate binders, vitamin D analogs.;Model 3: Adjusted for variables in Model 2 plus uric acid, platelet. Model 4: Adjusted for variables in Model 3 plus white blood cell,monocyte count,lymphocyte count.
(DOCX)

## References

[1] Stevens PE, Ahmed SB, Carrero JJ, Foster B, Francis A, Hall RK, et al. Kdigo 2024 clinical practice guideline for the evaluation and management of chronic kidney disease. Kidney Int. 2024;105:S117-314. doi: 10.1016/j.kint.2023.10.018 38490803

[2] Al-Jaishi AA, Oliver MJ, Thomas SM, Lok CE, Zhang JC, Garg AX, et al. Patency rates of the arteriovenous fistula for hemodialysis: a systematic review and meta-analysis. Am J Kidney Dis. 2014;63(3):464–78. doi: 10.1053/j.ajkd.2013.08.023 24183112

[3] Wang Z, Gui Z, Zhang L, Wang Z. Advances in the mechanisms of vascular calcification in chronic kidney disease. J Cell Physiol. 2025;240(1):e31464. doi: 10.1002/jcp.31464 39392232

[4] Zhang H, Lu H, Li W, Jiang G, Zou H, Expert Group of Nephrology Branch of China Academy of Chronic Disease Urology Nephrology and Blood Purification Commission of China Medical Education Association. Expert consensus on the establishment and maintenance of native arteriovenous fistula. Chronic Dis Transl Med. 2021;7:235–53. 34786543 10.1016/j.cdtm.2021.05.002 PMC8579016

[5] Vachharajani TJ. Diagnosis of arteriovenous fistula dysfunction. Semin Dial. 2012;25(4):445–50. doi: 10.1111/j.1525-139X.2012.01094.x 22694731

[6] Zhou W, Simic P, Zhou IY, Caravan P, Vela Parada X, Wen D, et al. Kidney glycolysis serves as a mammalian phosphate sensor that maintains phosphate homeostasis. J Clin Invest. 2023;133(8):e164610. doi: 10.1172/JCI164610 36821389 PMC10104895

[7] Erben RG. Physiological actions of fibroblast growth factor-23. Frontiers in Endocrinology. 2018;9:267. 29892265 10.3389/fendo.2018.00267 PMC5985418

[8] Rana S, Lemoine E, Granger JP, Karumanchi SA. Preeclampsia: pathophysiology, challenges, and perspectives. Circ Res. 2019;124:1094–112. 30920918 10.1161/CIRCRESAHA.118.313276

[9] Zhao X, Ma C, Gan L, Hou FF, Liang X, Chen X, et al. Current clinical profiles for Chinese hemodialysis patients. Sci Rep. 2025;15(1):28428. doi: 10.1038/s41598-025-11951-6 40760064 PMC12322052

[10] Zhan Y, He X, Hong D, Wang L, Li G. The current status of chronic kidney disease-mineral and bone disorder management in China. Sci Rep. 2022;12(1):16694. doi: 10.1038/s41598-022-20790-8 36202866 PMC9537533

[11] Zhang W, Du Q, Xiao J, Bi Z, Yu C, Ye Z, et al. Modification and Validation of the Phosphate Removal Model: A Multicenter Study. Kidney Blood Press Res. 2021;46(1):53–62. doi: 10.1159/000511375 33477164

[12] Li L, Xu R-F, He N, Hu T, Gao W-N, Wang X-F, et al. Research progress on measurement methods and evaluation of the hemodialysis adequacy index Kt/V. Ther Apher Dial. 2025;29(1):3–11. doi: 10.1111/1744-9987.14217 39387225

[13] Liu Z-H, Yu X-Q, Yang J-W, Jiang A-L, Liu B-C, Xing C-Y, et al. Prevalence and risk factors for vascular calcification in Chinese patients receiving dialysis: baseline results from a prospective cohort study. Curr Med Res Opin. 2018;34(8):1491–500. doi: 10.1080/03007995.2018.1467886 29672176

[14] Bai W, Li J, Liu J. Serum phosphorus, cardiovascular and all-cause mortality in the general population: A meta-analysis. Clin Chim Acta. 2016;461:76–82. doi: 10.1016/j.cca.2016.07.020 27475981

[15] Zhang H, Li G, Yu X, Yang J, Jiang A, Cheng H, et al. Progression of vascular calcification and clinical outcomes in patients receiving maintenance dialysis. JAMA Netw Open. 2023;6(5):e2310909. doi: 10.1001/jamanetworkopen.2023.10909 37126347 PMC10152309

[16] Li Y, Cui W, Wang J, Zhang C, Luo T. Factors associated with dysfunction of autogenous arteriovenous fistula in patients with maintenance hemodialysis: a retrospective study. Ann Palliat Med. 2021;10(4):4047–54. doi: 10.21037/apm-20-2196 33832310

[17] Zhou M, Lu FP. Effect of hyperphosphatemia on patency rate of arteriovenous fistula of patients with late fistula dysfunction/failure after reoperation. Zhonghua Yi Xue Za Zhi. 2018;98(42):3406–10. doi: 10.3760/cma.j.issn.0376-2491.2018.42.006 30440134

[18] Zhang F, Li J, Yu J, Jiang Y, Xiao H, Yang Y, et al. Risk factors for arteriovenous fistula dysfunction in hemodialysis patients: a retrospective study. Sci Rep. 2023;13(1):21325. doi: 10.1038/s41598-023-48691-4 38044365 PMC10694134

[19] Paloian NJ, Giachelli CM. A current understanding of vascular calcification in CKD. Am J Physiol Renal Physiol. 2014;307(8):F891-900. doi: 10.1152/ajprenal.00163.2014 25143458 PMC4200295

[20] Giachelli CM. Vascular calcification: in vitro evidence for the role of inorganic phosphate. J Am Soc Nephrol. 2003;14(9 Suppl 4):S300-4. doi: 10.1097/01.asn.0000081663.52165.66 12939385

[21] Wu M, Rementer C, Giachelli CM. Vascular calcification: an update on mechanisms and challenges in treatment. Calcif Tissue Int. 2013;93:365–73. 23456027 10.1007/s00223-013-9712-z PMC3714357

[22] Shanahan CM, Crouthamel MH, Kapustin A, Giachelli CM. Arterial calcification in chronic kidney disease: key roles for calcium and phosphate. Circ Res. 2011;109(6):697–711. doi: 10.1161/CIRCRESAHA.110.234914 21885837 PMC3249146

[23] Voelkl J, Lang F, Eckardt K-U, Amann K, Kuro-O M, Pasch A, et al. Signaling pathways involved in vascular smooth muscle cell calcification during hyperphosphatemia. Cell Mol Life Sci. 2019;76(11):2077–91. doi: 10.1007/s00018-019-03054-z 30887097 PMC6502780

[24] Voelkl J, Tuffaha R, Luong TTD, Zickler D, Masyout J, Feger M, et al. Zinc Inhibits Phosphate-Induced Vascular Calcification through TNFAIP3-Mediated Suppression of NF-κB. J Am Soc Nephrol. 2018;29(6):1636–48. doi: 10.1681/ASN.2017050492 29654213 PMC6054342

[25] Gross P, Six I, Kamel S, Massy ZA. Vascular toxicity of phosphate in chronic kidney disease: beyond vascular calcification . Circ J. 2014;78(10):2339–46. doi: 10.1253/circj.cj-14-0735 25077548

[26] Six I, Maizel J, Barreto FC, Rangrez AY, Dupont S, Slama M, et al. Effects of phosphate on vascular function under normal conditions and influence of the uraemic state. Cardiovasc Res. 2012;96(1):130–9. doi: 10.1093/cvr/cvs240 22822101

[27] Di Marco GS, Hausberg M, Hillebrand U, Rustemeyer P, Wittkowski W, Lang D, et al. Increased inorganic phosphate induces human endothelial cell apoptosis in vitro. Am J Physiol Renal Physiol. 2008;294(6):F1381-7. doi: 10.1152/ajprenal.00003.2008 18385273

[28] Shuto E, Taketani Y, Tanaka R, Harada N, Isshiki M, Sato M, et al. Dietary Phosphorus Acutely Impairs Endothelial Function. J Am Soc Nephrol. 2009;20:1504-1512. 19406976 10.1681/ASN.2008101106 PMC2709683

[29] Stevens KK, Denby L, Patel RK, Mark PB, Kettlewell S, Smith GL, et al. Deleterious effects of phosphate on vascular and endothelial function via disruption to the nitric oxide pathway. Nephrol Dial Transplant. 2017;32(10):1617–27. doi: 10.1093/ndt/gfw252 27448672 PMC5837731

[30] Perticone M, Maio R, Sciacqua A, Cimellaro A, Andreucci M, Tripepi G, et al. Serum phosphorus levels are associated with endothelial dysfunction in hypertensive patients. Nutr Metab Cardiovasc Dis. 2016;26(8):683–8. doi: 10.1016/j.numecd.2016.02.003 27105871

[31] Nishida Y, Taketani Y, Yamanaka-Okumura H, Imamura F, Taniguchi A, Sato T, et al. Acute effect of oral phosphate loading on serum fibroblast growth factor 23 levels in healthy men. Kidney Int. 2006;70(12):2141–7. doi: 10.1038/sj.ki.5002000 17063170

[32] Morrissey JH. Polyphosphate: a link between platelets, coagulation and inflammation. Int J Hematol. 2012;95(4):346–52. doi: 10.1007/s12185-012-1054-5 22477540 PMC3361507

[33] Abbasian N, Harper MT. High extracellular phosphate increases platelet polyphosphate content. Platelets. 2021;32(7):992–4. doi: 10.1080/09537104.2020.1817358 32892685 PMC8437092

[34] Abbasian N, Burton JO, Herbert KE, Tregunna B-E, Brown JR, Ghaderi-Najafabadi M, et al. Hyperphosphatemia, phosphoprotein phosphatases, and microparticle release in vascular endothelial cells. J Am Soc Nephrol. 2015;26(9):2152–62. doi: 10.1681/ASN.2014070642 25745026 PMC4552113

[35] Carlström M, Weitzberg E, Lundberg JO. Nitric oxide signaling and regulation in the cardiovascular system: recent advances. Pharmacol Rev. 2024;76(6):1038–62. doi: 10.1124/pharmrev.124.001060 38866562

[36] Jean G, Bresson E, Lorriaux C, Mayor B, Hurot J-M, Deleaval P, et al. Increased levels of serum parathyroid hormone and fibroblast growth factor-23 are the main factors associated with the progression of vascular calcification in long-hour hemodialysis patients. Nephron Clin Pract. 2012;120(3):c132-8. doi: 10.1159/000334424 22584463

[37] Raggi P, Chertow GM, Torres PU, Csiky B, Naso A, Nossuli K, et al. The ADVANCE study: a randomized study to evaluate the effects of cinacalcet plus low-dose vitamin D on vascular calcification in patients on hemodialysis. Nephrol Dial Transplant. 2011;26(4):1327–39. doi: 10.1093/ndt/gfq725 21148030

[38] Carrillo-López N, Panizo S, Alonso-Montes C, Martínez-Arias L, Avello N, Sosa P, et al. High-serum phosphate and parathyroid hormone distinctly regulate bone loss and vascular calcification in experimental chronic kidney disease. Nephrol Dial Transplant. 2019;34(6):934–41. doi: 10.1093/ndt/gfy287 30189026

[39] Wu G-Y, Xu B-D, Wu T, Wang X-Y, Wang T-X, Zhang X, et al. Correlation between serum parathyroid hormone levels and coronary artery calcification in patients without renal failure. Biomed Rep. 2016;5(5):601–6. doi: 10.3892/br.2016.761 27882224 PMC5103683

[40] Cheng Z-Y, Ye T, Ling Q-Y, Wu T, Wu G-Y, Zong G-J. Parathyroid hormone promotes osteoblastic differentiation of endothelial cells via the extracellular signal-regulated protein kinase 1/2 and nuclear factor-κB signaling pathways. Exp Ther Med. 2018;15(2):1754–60. doi: 10.3892/etm.2017.5545 29434762 PMC5774494

[41] Liu Y, Wu Y, Diao Z, Guo W, Liu W. Resveratrol inhibits parathyroid hormone-induced apoptosis in human aortic smooth muscle cells by upregulating sirtuin 1. Ren Fail. 2019;41(1):401–7. doi: 10.1080/0886022X.2019.1605296 31106631 PMC6534218

[42] Yang H, Curinga G, Giachelli CM. Elevated extracellular calcium levels induce smooth muscle cell matrix mineralization in vitro. Kidney Int. 2004;66(6):2293–9. doi: 10.1111/j.1523-1755.2004.66015.x 15569318

[43] Zhao H, Liu G, Wang Q, Ding L, Cai H, Jiang H, et al. Effect of ghrelin on human endothelial cells apoptosis induced by high glucose. Biochem Biophys Res Commun. 2007;362(3):677–81. doi: 10.1016/j.bbrc.2007.08.021 17719561

[44] Li H, Qian F, Liu H, Zhang Z. Elevated uric acid levels promote vascular smooth muscle cells (VSMC) proliferation via an Nod-Like Receptor Protein 3 (NLRP3)-inflammasome-dependent mechanism. Med Sci Monit. 2019;25:8457–64. 31707403 10.12659/MSM.916667 PMC6865250

[45] Yang X, Gu J, Lv H, Li H, Cheng Y, Liu Y, et al. Uric acid induced inflammatory responses in endothelial cells via up-regulating(pro)renin receptor. Biomed Pharmacother. 2019;109:1163–70. doi: 10.1016/j.biopha.2018.10.129 30551366

[46] Hammer HB, Rollefstad S, Semb AG, Jensen G, Karoliussen LF, Terslev L, et al. Urate crystal deposition is associated with inflammatory markers and carotid artery pathology in patients with intercritical gout: results from the NOR-Gout study. RMD Open. 2022;8(2):e002348. doi: 10.1136/rmdopen-2022-002348 35863863 PMC9310249

[47] Yu S, Hong Q, Wang Y, Hou K, Wang L, Zhang Y, et al. High concentrations of uric acid inhibit angiogenesis via regulation of the Krüppel-like factor 2-vascular endothelial growth factor-A axis by miR-92a. Circ J. 2015;79(11):2487–98. doi: 10.1253/circj.CJ-15-0283 26299712

[48] Yang H, Bai W, Gao L, Jiang J, Tang Y, Niu Y, et al. Mangiferin alleviates hypertension induced by hyperuricemia via increasing nitric oxide releases. J Pharmacol Sci. 2018;137(2):154–61. doi: 10.1016/j.jphs.2018.05.008 29934052

