# Supplementary figures and images for: Hyperphosphatemia as a potential risk factor for arteriovenous fistula dysfunction: A retrospective study in hemodialysis patients
Source: PLoS One. 2025 Oct 30;20(10):e0335599. doi: 10.1371/journal.pone.0335599 (PMC12574839; doi:10.1371/journal.pone.0335599)

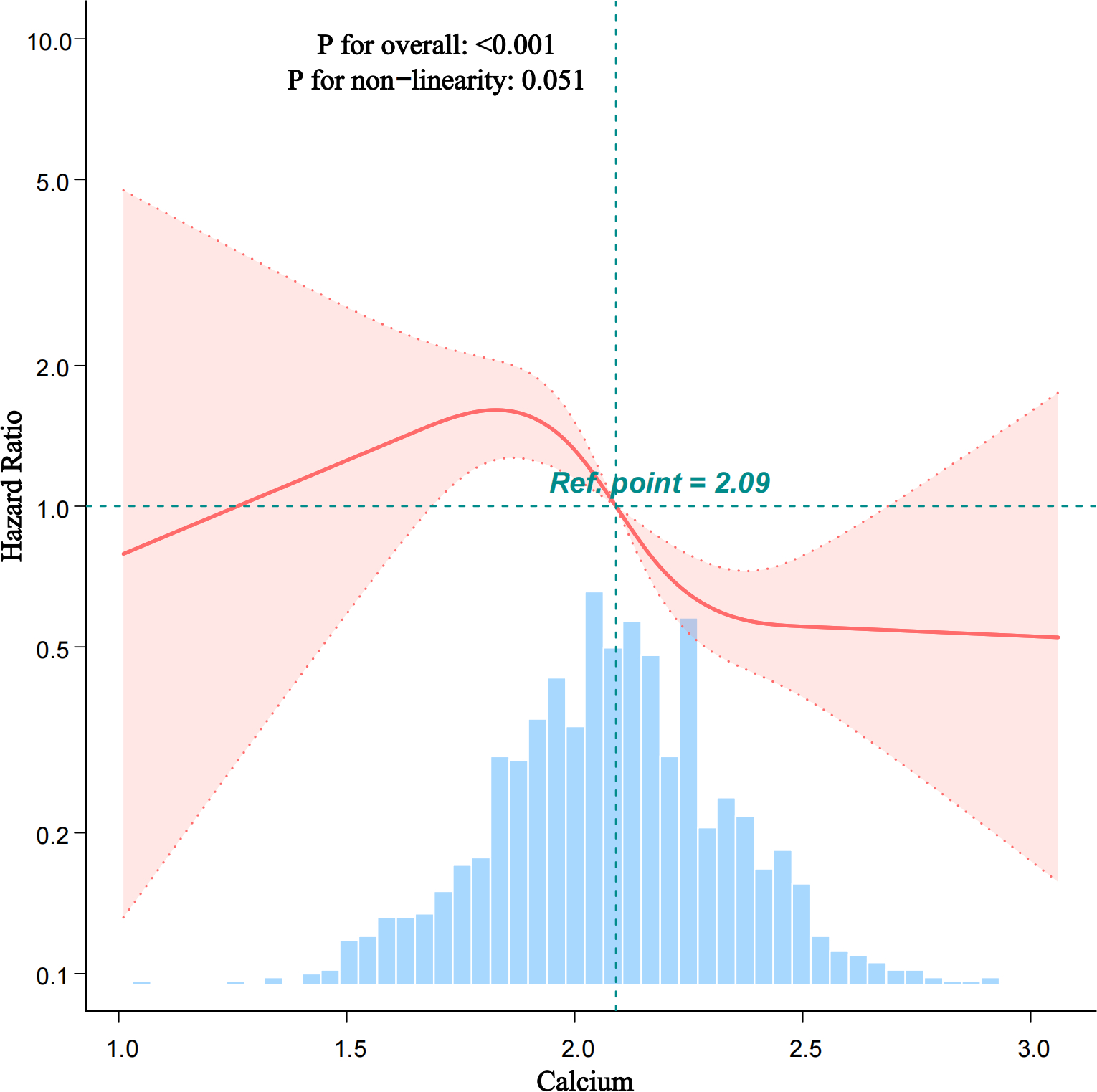

Supplement: S1 Fig — The solid red line represents the hazard ratio (HR), and the shaded area indicates the 95% confidence interval. (TIF) [file pone.0335599.s001.tif]
