# Supplementary material for: Hyperphosphatemia as a potential risk factor for arteriovenous fistula dysfunction: A retrospective study in hemodialysis patients
Source: PLoS One. 2025 Oct 30;20(10):e0335599. doi: 10.1371/journal.pone.0335599 (PMC12574839; doi:10.1371/journal.pone.0335599)
Supplement: S1 Table — Model 1: Unadjusted.; Model 2: Adjusted for sex, age, history of hypertension, diabetes, cardiovascular disease, parathyroid hormone, phosphorus, use of phosphate binders, vitamin D analogs.;Model 3: Adjusted for variables in Model 2 plus uric acid, platelet. Model 4: Adjusted for variables in Model 3 plus white blood cell. (DOCX) [file pone.0335599.s002.docx]

**Table 1S.** Hazard ratio for arteriovenous fistula dysfunction events according to the baseline of calcium

| Mode l | C_1_ (＜2.52 mmol/L) | C_2_ (≥ 2.52 mmol/L) | |
| --- | --- | --- | --- |
|  |  | HR（95%CI） | P |
| Model 1 | Reference | 0.59 (0.31~1.12) | 0.106 |
| Model 2 | Reference | 0.57 (0.31~1.04) | 0.065 |
| Model 3 | Reference | 0.55 (0.3~1.01) | 0.056 |
| Model 4 | Reference | 0.56 (0.3~1.02) | 0.059 |

Model 1: Unadjusted.; Model 2: Adjusted for sex, age, history of hypertension, diabetes, cardiovascular disease, parathyroid hormone, phosphorus, use of phosphate binders, vitamin D analogs.;Model 3: Adjusted for variables in Model 2 plus uric acid, platelet. Model 4: Adjusted for variables in Model 3 plus white blood cell.
