# Supplementary material for: Hyperphosphatemia as a potential risk factor for arteriovenous fistula dysfunction: A retrospective study in hemodialysis patients
Source: PLoS One. 2025 Oct 30;20(10):e0335599. doi: 10.1371/journal.pone.0335599 (PMC12574839; doi:10.1371/journal.pone.0335599)
Supplement: S2 Table — Model 1: Unadjusted.; Model 2: Adjusted for sex, age, history of hypertension, diabetes, cardiovascular disease, parathyroid hormone, phosphorus, use of phosphate binders, vitamin D analogs.;Model 3: Adjusted for variables in Model 2 plus uric acid, platelet. Model 4: Adjusted for variables in Model 3 plus white blood cell,monocyte count,lymphocyte count. (DOCX) [file pone.0335599.s003.docx]

**Table 2S. Hazard ratio for arteriovenous fistula dysfunction events according to the baseline of calcium(cut-off 2.09 mmol/L)**

| Mode l | C_3_ (＜2.09 mmol/L) | C_4_ (≥ 2.09 mmol/L) | |
| --- | --- | --- | --- |
|  |  | HR（95%CI） | P |
| Model 1 | Reference | 0.39 (0.29~0.53) | <0.001 |
| Model 2 | Reference | 0.4 (0.29~0.54) | <0.001 |
| Model 3 | Reference | 0.37 (0.27~0.52) | <0.001 |
| Model 4 | Reference | 0.39 (0.29~0.53) | <0.001 |

Model 1: Unadjusted.; Model 2: Adjusted for sex, age, history of hypertension, diabetes, cardiovascular disease, parathyroid hormone, phosphorus, use of phosphate binders, vitamin D analogs.;Model 3: Adjusted for variables in Model 2 plus uric acid, platelet. Model 4: Adjusted for variables in Model 3 plus white blood cell,monocyte count,lymphocyte count.
